# Supplementary material for: Calciprotein Particles Cause Endothelial Dysfunction under Flow
Source: Int J Mol Sci. 2020 Nov 20;21(22):8802. doi: 10.3390/ijms21228802 (PMC7699979; doi:10.3390/ijms21228802)
Supplement: Supplementary file 1 [file ijms-21-08802-s001.zip › Legends to Supplementary Figures.pdf]

Legends to Supplementary Figures

Supplementary Figure 1. Bar graphs of the: **(a)** Western blotting measurements of cell adhesion molecules VCAM1 and ICAM1 as compared to the expression of three EC housekeeping proteins (vimentin, CD31 and VE-cadherin) in HCAEC and HITAEC co-incubated with PBS, MPP, CPP-P or CPP-S in a flow system for 4 hours. Bars represent the measurements of the sample pooled from three sequential flow culture runs (i.e., three biological replicates). Green, blue, orange and red colours mean PBS, MPP, CPP-P and CPP-S groups, respectively; **(b)** Gene expression analysis (by means of RT-qPCR) of *VCAM1*, *ICAM1*, and *SELE* genes in HCAEC and HITAEC co-incubated with PBS, MPP, CPP-P or CPP-S in a flow system for 4 hours. Bars represent the measurements of the sample pooled from three sequential flow culture runs (i.e., three biological replicates). Green, blue, orange and red colours mean PBS, MPP, CPP-P and CPP-S groups, respectively; **(c)** Western blotting measurement of Vcam1 as compared to the expression of three EC housekeeping proteins (Cd31, Gapdh, and histone H3) in the endothelial lysate collected from the descending aorta and aortic arch of Wistar rats which received consecutive tail vein injections of CPP-P, CPP-S or 0.9% NaCl (10 daily injections). Bars represent the measurements obtained from three experimental animals per group. P values provided above boxes, Kruskal-Wallis test with post hoc false discovery rate correction by two-stage linear step-up procedure of Benjamini, Krieger and Yekutieli. Green, orange, and red colours mean NaCl, CPP-P and CPP-S groups, respectively; **(d)** Gene expression analysis (by means of RT-qPCR) of *Vcam1* and *Icam1* genes in the endothelial lysate collected from the descending aorta and aortic arch of Wistar rats which received consecutive tail vein injections of CPP-P, CPP-S or 0.9% NaCl (10 daily injections). Bars represent the measurements obtained from three experimental animals per group. P values provided above boxes, Kruskal-Wallis test with post hoc false discovery rate correction by two-stage linear step-up procedure of Benjamini, Krieger and Yekutieli. Green, orange, and red colours mean NaCl, CPP-P and CPP-S groups, respectively. HCAEC – human coronary artery endothelial cells, HITAEC – human internal thoracic artery endothelial cells, PBS – phosphate-buffered saline, MPP – magnesiprotein particles, CPP-P – primary calciprotein particles, CPP-S – secondary calciprotein particles, RT-qPCR – reverse transcription quantitative polymerase chain reaction, VCAM1 – vascular cell adhesion molecule 1, ICAM1 – intercellular cell adhesion molecule 1, CD31 – cluster of differentiation 31, VE-cadherin – vascular endothelial cadherin, SELE – E-selectin, Gapdh - glyceraldehyde 3-phosphate dehydrogenase.

Supplementary Figure 2. Bar graphs of the: **(a)** Western blotting measurements of EndoMT transcription factors Snail and Slug and EndoMT marker N-cadherin as compared to the expression of vimentin, CD31 and VE-cadherin in HCAEC and HITAEC co-incubated with PBS, MPP, CPP-P or CPP-S in a flow system for 4 hours. Bars represent the measurements of the sample pooled from three sequential flow culture runs (i.e., three biological replicates). Green, blue, orange and red colours mean PBS, MPP, CPP-P and CPP-S groups, respectively; **(b)** Gene expression analysis (by means of RT-qPCR) of *SNAI1*, *SNAI2*, *TWIST1*, *ZEB1*, *CDH2*, *ACTA2*, *KDR*, *PECAM1* and *CDH5* genes in HCAEC and HITAEC co-incubated with PBS, MPP, CPP-P or CPP-S in a flow system for 4 hours. Bars represent the measurements of the sample pooled from three sequential flow culture runs (i.e., three biological replicates). Green, blue, orange and red colours mean PBS, MPP, CPP-P and CPP-S groups, respectively; **(c)** Western blotting measurements of EndoMT transcription factors Snail, Slug and Twist1 and α-smooth muscle actin as compared to the expression of Cd31, Gapdh and histone H3 in the endothelial lysate collected from the descending aorta and aortic arch of Wistar rats which received consecutive tail vein injections of CPP-P, CPP-S or 0.9% NaCl (10 daily injections). Bars represent the measurements obtained from three experimental animals per group. P values provided above boxes, Kruskal-Wallis test with post hoc false discovery rate correction by two-stage linear step-up procedure of Benjamini, Krieger and Yekutieli. Green, orange, and red colours mean NaCl, CPP-P and CPP-S groups, respectively; **(d)** Gene expression analysis (by means of RT-qPCR) of *Twist1*, *Zeb1*, *Cdh2*, *Kdr*, *Pecam1*, and *Cdh5* genes in the endothelial lysate collected from the descending aorta and aortic arch of Wistar rats which received consecutive tail vein injections of CPP-P, CPP-S, or 0.9% NaCl (10 daily injections). Bars represent the measurements obtained from three experimental animals per group. P values provided above boxes, Kruskal-Wallis test with post hoc false discovery rate correction by two-stage linear step-up procedure of Benjamini, Krieger and Yekutieli. Green, orange, and red colours mean NaCl, CPP-P

and CPP-S groups, respectively. HCAEC – human coronary artery endothelial cells, HITAEC – human internal thoracic artery endothelial cells, PBS – phosphate-buffered saline, MPP – magnesiprotein particles, CPP-P – primary calciprotein particles, CPP-S – secondary calciprotein particles, RT-qPCR – reverse transcription quantitative polymerase chain reaction, N-cadherin – neural cadherin, CD31 – cluster of differentiation 31, VE-cadherin – vascular endothelial cadherin, SNAI – Snail family transcriptional repressor, TWIST – Twist family basic helix-loop-helix transcription factor, ZEB – Zinc finger E-box binding homeobox, CDH – cadherin, ACTA – actin alpha, KDR – kinase insert domain receptor, PECAM – platelet endothelial cell adhesion molecule, αSma – α-smooth muscle actin, Gapdh - glyceraldehyde 3-phosphate dehydrogenase.

Supplementary Figure 3. Bar graphs of the: **(a)** Western blotting measurements of mechanosensitive transcription factors KLF2, KLF4, NRF2, YAP1 and TAZ and phosphorylated forms of YAP1 and TAZ as compared to the expression of vimentin, CD31 and VE-cadherin in HCAEC and HITAEC co-incubated with PBS, MPP, CPP-P or CPP-S in a flow system for 4 hours. Bars represent the measurements of the sample pooled from three sequential flow culture runs (i.e., three biological replicates). Green, blue, orange and red colours mean PBS, MPP, CPP-P and CPP-S groups, respectively; **(b)** Gene expression analysis (by means of RT-qPCR) of *KLF2*, *KLF4*, *NFE2L2*, *YAP1*, and *WWTR1* genes in HCAEC and HITAEC co-incubated with PBS, MPP, CPP-P or CPP-S in a flow system for 4 hours and of the respective genes (*Klf2*, *Klf4*, *Nfe2l2*, *Yap1*, and *Wwtr1*) in the endothelial lysate collected from the descending aorta and aortic arch of Wistar rats which received consecutive tail vein injections of CPP-P, CPP-S or 0.9% NaCl (10 daily injections). For HCAEC and HITAEC, bars represent the measurements of the sample pooled from three sequential flow culture runs (i.e., three biological replicates). Green, blue, orange and red colours mean PBS, MPP, CPP-P and CPP-S groups, respectively. For descending aorta and aortic arch, bars represent the measurements obtained from three experimental animals per group. P values provided above boxes, Kruskal-Wallis test with post hoc false discovery rate correction by two-stage linear step-up procedure of Benjamini, Krieger and Yekutieli. Green, orange, and red colours mean NaCl, CPP-P and CPP-S groups, respectively; **(c)** Western blotting measurements of mechanosensitive transcription factors Klf2, Klf4, Nrf2, Yap1, Taz and phosphorylated forms of Yap1 and Taz as compared to the expression of Cd31, Gapdh and histone H3 in the endothelial lysate collected from the descending aorta and aortic arch of Wistar rats which received consecutive tail vein injections of CPP-P, CPP-S or 0.9% NaCl (10 daily injections). Bars represent the measurements obtained from three experimental animals per group. P values provided above boxes, Kruskal-Wallis test with post hoc false discovery rate correction by two-stage linear step-up procedure of Benjamini, Krieger and Yekutieli. Green, orange, and red colours mean NaCl, CPP-P and CPP-S groups, respectively. HCAEC – human coronary artery endothelial cells, HITAEC – human internal thoracic artery endothelial cells, PBS – phosphate-buffered saline, MPP – magnesiprotein particles, CPP-P – primary calciprotein particles, CPP-S – secondary calciprotein particles, RT-qPCR – reverse transcription quantitative polymerase chain reaction, KLF – Krüppel-like factor, NRF – nuclear factor erythroid 2–related factor, YAP – Yes-associated protein, pYAP – phosphorylated YAP, TAZ – transcriptional co-activator with PDZ binding motif, pTAZ – phosphorylated TAZ, CD31 – cluster of differentiation 31, VE-cadherin – vascular endothelial cadherin, NFE2L2 – nuclear factor erythroid 2 like 2, WWTR – WW domain containing transcription regulator, Gapdh - glyceraldehyde 3-phosphate dehydrogenase.
