# Supplementary figures and images for: Calciprotein Particles Cause Endothelial Dysfunction under Flow
Source: Int J Mol Sci. 2020 Nov 20;21(22):8802. doi: 10.3390/ijms21228802 (PMC7699979; doi:10.3390/ijms21228802)

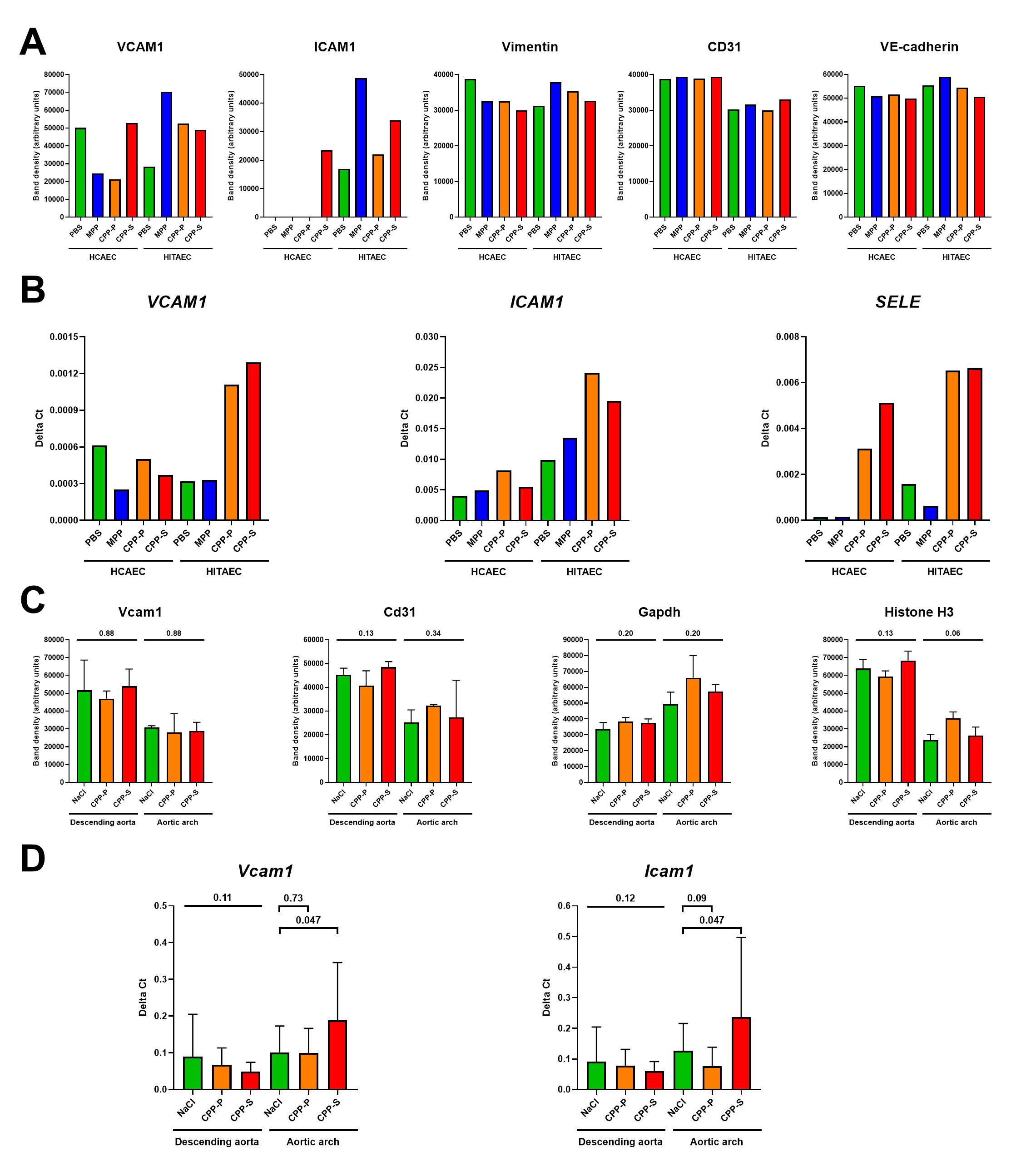

Supplement: Supplementary file 1 [file ijms-21-08802-s001.zip › Supplementary Figure 1.tif]

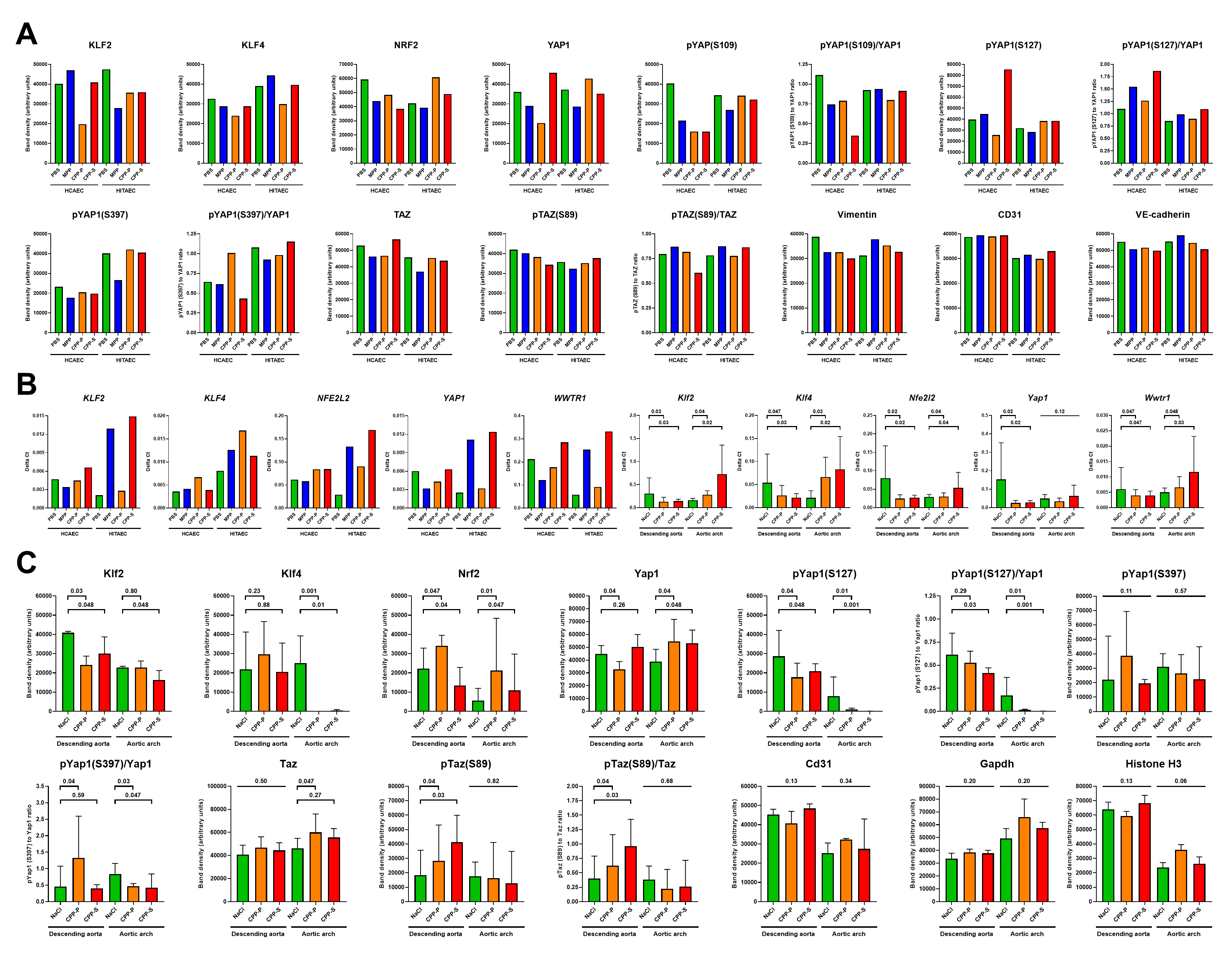

Supplement: Supplementary file 1 [file ijms-21-08802-s001.zip › Supplementary Figure 3.tif]

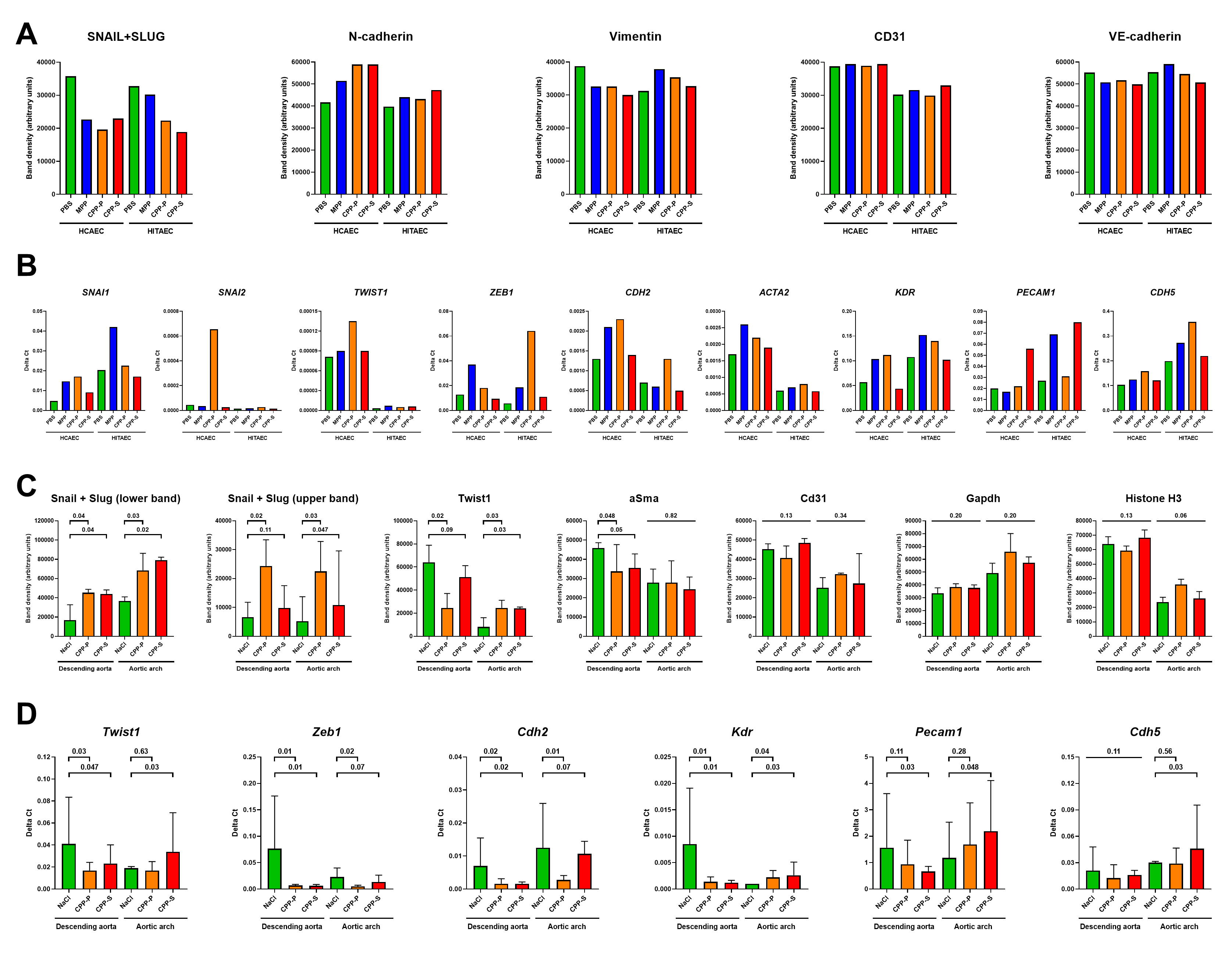

Supplement: Supplementary file 1 [file ijms-21-08802-s001.zip › Supplementary Figure 2.tif]
